# Illness perception among patients with chest pain and palpitations before and after negative cardiac evaluation

**DOI:** 10.1186/1751-0759-6-19

**Published:** 2012-09-27

**Authors:** Egil Jonsbu, Egil W Martinsen, Gunnar Morken, Torbjørn Moum, Toril Dammen

**Affiliations:** 1 Department of Psychiatry, More and Romsdal Hospital Trust, Molde, 6407, Norway; 2 Department of Neuroscience, Norwegian University of Science and Technology, Trondheim, Norway; 3 Institute of Clinical Medicine, Faculty of Medicine, University of Oslo, Oslo, 0318, Norway; 4 Division of Mental Health and Addiction, Oslo University Hospital, Oslo, N-0424, Norway; 5 Østmarka Department of Psychiatry, St Olavs University Hospital, Trondheim, Norway; 6 Institute of Basic Medical Sciences, Department of Behavioural Sciences in Medicine, Faculty of Medicine, University of Oslo, Oslo, 0317, Norway

**Keywords:** Non-cardiac chest pain, Benign palpitations, Negative cardiac evaluation, Psychosomatic medicine, Illness perception

## Abstract

**Background:**

Patients with chest pain or palpitations often have poor outcomes following a negative cardiac evaluation, with symptom persistence, limitations in everyday activities, and reduced health-related quality of life. The aims of this study were to evaluate illness perceptions before and after negative cardiac evaluations and measure the ability of a self-report questionnaire to predict outcomes.

**Methods:**

Patients (*N* = 138) referred for chest pain or palpitations to a cardiac outpatient clinic were assessed before and six months after a negative cardiac evaluation. In addition to Brief Illness Perception Questionnaire (BIPQ), all patients completed the Beck Depression Inventory and SF-36 Health Survey.

**Results:**

The emotional reactions to and understanding of symptoms had not improved six months after a negative cardiac evaluation. A stronger correlation between illness perceptions and health at follow-up than before the cardiac evaluation might explain the tendency for poor outcomes among these patients. Most of the eight BIPQ item scores before the negative cardiac evaluation were predictive of the outcome six months later. A single question asking about the perceived consequences of the complaints (BIPQ Item 1) rated before the cardiac evaluation was collapsed into a dichotomous variable with a cut-off at ≥4 which yields a sensitivity of 51%, a specificity of 85%, a positive predictive value of 71%, a negative predictive value of 69%, and an odds ratio of 5.7 (*r* = .38, p < .001) in predicting poor outcomes.

**Conclusions:**

Assessing illness perceptions is important in patients with negative cardiac tests for understanding and predicting outcomes.

## Background

Chest pain and palpitations are the two most common reasons for referral to a cardiologist [1]. Most of these patients suffer from non-cardiac chest pain and benign palpitations [2-4]. Little is known about the course of psychological symptoms from before to after negative cardiac evaluation. However, studies which have evaluated the long term outcome for these patients report poor outcome with worries about bodily sensations, avoidance of physical activity, and reduced health-related quality of life (HRQOL) [5-7]. McDonald et al. [8] reported that patients had a tendency toward more anxiety about the heart after a negative cardiac evaluation than before. In another study [5], patients reported significantly higher levels of fear about bodily symptoms and more depression six months after a negative cardiac evaluation compared with before the evaluation.

The perception of illness affects the way patients cope with their complaints and is important for outcome [9-12]. Broadbent et al. [13] showed that a four-session intervention aiming to change illness perceptions was associated with improved rates of return to work after a myocardial infarction (MI). Robertsen et al. [14] compared illness perceptions among non-cardiac and cardiac patients, who all were new attendees at a rapid access chest pain clinic. Patients with non-cardiac chest pain reported less understanding and personal control of their chest pain both before and after the cardiac evaluation than the cardiac patients. Patients with non-cardiac chest pain or benign palpitations seem to have similar psychological characteristics and outcomes [2,5]. However, to the best of our knowledge, no study has evaluated illness perceptions among patients with benign palpitations.

Illness perceptions have been assessed by The Brief Illness Perception Questionnaire (BIPQ) in several patient groups [15]. However, there is limited knowledge about how BIPQ item scores change following a negative cardiac evaluation and to what extent illness perception may explain the poor outcome in terms of significant complaints for a high proportion of these patients.

Even though the aetiology of non-cardiac chest pain and benign palpitations is multifactorial, psychological treatments are useful [16,17]. Thus there is a need to identify patients who could benefit from such treatment. In a recent study [5] it was found that a score on the Beck Depression Inventory (BDI) of five or above, rated before the cardiac evaluation, served reasonably well as a screening instrument for identifying patients in need of psychological treatment for their complaints six months later. An ideal screening test for use in addition to cardiac evaluation, should be simpler than the BDI with 21 items. Donkin et al. [18] reported that illness perceptions as measured by the BIPQ among patients before undergoing a normal cardiac stress test, predicted reassurance regarding cardiac health one month later. That study did not evaluate whether the BIPQ predicted depression or HRQOL, or whether the test could serve as a screening tool for identifying patients with poor outcomes.

We aimed to evaluate the similarities and differences in illness perceptions between patients with chest pain and palpitations and changes in illness perceptions from before to six months after a negative cardiac evaluation. Other aims were to analyse the relations between illness perceptions and depression and HRQOL before and six months after the cardiac evaluation and to evaluate to what extent illness perceptions before the cardiac evaluation could predict outcomes six months later.

## Methods

### Patients

Consecutive patients referred to the cardiac outpatient unit at the Molde Hospital in Norway between May 2006 and May 2007 for the evaluation of chest pain or palpitations were asked to participate. The outpatient clinic receives all referrals in a catchment area of about 75,000 inhabitants. The head of the cardiac unit screened all referrals. The inclusion criteria were: (1) referral for a main complaint of chest pain or palpitations; (2) age 18–65 years; and (3) ability to understand and write Norwegian. The exclusion criteria were: (1) mental retardation; (2) psychosis; or (3) organic heart disease confirmed by a cardiologist. Of the 219 consecutive patients, 21 cancelled both the cardiac and the psychiatric evaluations and 36 did not want to participate in the study. A total of 162 patients participated in the psychiatric and cardiac evaluations at admission. Of these, eight were excluded, six because of coronary heart disease confirmed by the cardiac evaluation (five in the chest pain group and one in the palpitations group), one because of lack of Norwegian language competency, and one because of mental retardation. No arrhythmias in need of treatment were detected.

Furthermore, of the 154 patients who fulfilled the criteria at attendance, 138 (90%) responded to mailed questionnaires at the six-month follow-up: 95/107 (89%) in the chest pain group and 43/47 (91%) in the palpitations group. These 138 patients were included in analyses at attendance and at six months follow-up.

The patients who did not participate at follow-up did not differ significantly at attendance from the participants who responded in terms of gender, age, prevalence of psychiatric disorders, or scores for any variable at attendance relevant to the outcome.

### Cardiac evaluation

The patients referred for chest pain underwent a standard bicycle stress test. If the results were consistent with or doubt arose regarding the presence of coronary heart disease, the patients were referred for myocardial scintigraphy or coronary angiography. The patients referred for palpitations were monitored with 24-h Holter monitoring and seven days of electrocardiography (ECG) monitoring (R-test) or if necessary a bicycle stress test. The procedure is described in detail elsewhere [4].

### Demographic data and psychiatric disorders

Gender, age, marital status, education, work status, duration of symptoms, numbers of days on sick leave during the three months prior to evaluation, and any psychiatric disorders were registered at attendance (Table 1). Psychiatric disorders were assessed by the Structured Clinical Interview for DSM-IV axis I disorders (SCID-I) [19]. The first author, who is an experienced psychiatrist and trained in the use of this instrument, performed the interviews. For current diagnoses, the criteria had to be met within one month before the interview. All patients were informed about the results of the psychiatric evaluation.

**Table 1:** Demographic and clinical data before the cardiac evaluation

	**Total**	**Chest pain**	**Palpitations**	**p**
	***N*** = **138**	***n*** = **95**	***n*** = **43**	
Women	78 (56%)	47 (50%)	31 (72%)	.01^a^
Age, mean (SD)	50 (10.7)	53 (9.4)	44 (11.0)	<.001^b^
Married/cohabiting	107 (78%)	77 (81%)	30 (70%)	ns
Vocational school/university	96 (70%)	66 (70%)	30 (70%)	ns
Main source of income in the past 6 months:				
Work	88 (64%)	58 (61%)	30 (70%)	ns
Sickness benefit	39 (28%)	29 (31%)	10 (23%)	ns
Other	11 (8%)	8 (8%)	3 (7%)	ns
Duration of symptoms in months before evaluation; median (range)	9 (1–420)	9.5 (1–420)	6.5 (2–360)	ns
Days on sick leave in the past 3 months; median (range)	7.5 (0–90)	8 (0–90)	2.5 (0–90)	ns
Current psychiatric disorders	53 (38%)	36 (38%)	17 (40%)	ns

^a^Chi-squared test.

^b^Student’s *t*-test.

ns, not significant.

### Brief illness perception questionnaire

The BIPQ was designed to provide a rapid assessment of a patient’s personal perception of his or her illness [15]. BIPQ consists of eight items related to illness perception, rated on a 0–10 scale. In addition, patients are asked to identify the three most important factors that they believe have caused their illness. The eight aspects of illness perceptions are: 1. consequences (“How much does your illness affect your life?”); 2. timeline (“How long do you think your illness will continue?”); 3. personal control (“How much control do you feel you have over your illness?”); 4. treatment control (“How much do you think your treatment can help your illness?”); 5. symptom frequency (“How much do you experience symptoms from your illness?”); 6. illness concern (“How concerned are you about your illness?”); 7. understanding (“How well do you feel you understand your illness?”); and 8. emotional effect (“How much does your illness affect you emotionally? e.g., does it make you angry, scared, upset, or depressed?”). The questions 3, 4 and 7 are reversed, that means higher score is supposed to be beneficial. The BIPQ shows good test–retest reliability [15]. We applied an approved Norwegian translation of the BIPQ (Sivertsen and Havik 2004). In order to adapt the questionnaire to the present non-cardiac chest pain and benign palpitations, the word “illness” was replaced with “complaints”. It was emphasized that patients should relate “complaints” to the main reason for referral (chest pain or palpitations).

### Depression

The BDI [20] measures the level of depression and comprises 21 items rated on a 0–3 scale. The questionnaire has sound psychometric properties, it is widely used clinically and in research, and has also previously been used in studies among non-cardiac chest pain and benign palpitations patients [2].

### Health-related quality of life (SF-36)

The 36-item SF-36 Health Survey [21] measures the patient’s perception of HRQOL. The scores can be converted into eight domains (physical functioning, physical role limitations, bodily pain, general health perception, vitality, social function, emotional role limitations, and mental health) or two dimensions, the Physical Component Scale (PCS) and the Mental Component Scale (MCS) [22]. To limit the number of analyses, the two-dimension approach was used in the present study.

### Frequency of symptoms

The frequency of symptoms was rated as: 1, daily; 2, weekly or more often; 3, rarely but sometimes; or 4, no symptoms in the past six months. For patients who reported both chest pain and palpitations, the highest frequency score was used in the statistical analyses.

### Consequences of chest pain and palpitations

The impact of chest pain or palpitations on the domains of family, social, and work life were recorded separately as: 1, high impact; 2, moderate impact; 3, some impact; and 4, no impact. The highest score (the domain most affected) among the three was used. Avoidance of physical activity was assessed by the question: “Do you avoid physical activity because of worries about the heart?” The response categories were: 1, often; 2, now and then; 3, rarely but sometimes; and 4, never.

### Clinically significant complaints

Clinically significant complaints consisted of a combination of the variables Frequency of symptoms and Consequences of chest pain and palpitations. It was defined as at least one of the following: (1) at least weekly symptoms; (2) at least moderate impact on family life, social life, or work; or (3) at least avoiding physical activity now and then because of worries about the heart. Clinically significant complaints were categorized as present or absent, and 43% (60/138) fulfilled these criteria [5].

### Procedure

The inquiry and information about participation in the study were mailed together with the appointment time at the cardiac outpatient clinic. The psychiatric evaluation and assessments at attendance were performed before the cardiac evaluation; thus, both the interviewer and the patients were blind to the results of the cardiac evaluation. The questionnaires BIPQ, BDI and SF-36 were rated before and six months after the cardiac evaluation. Data for Clinically significant complaints were collected six months after the cardiac evaluation. All information about the patients at the six-month follow-up was collected by mail.

### Ethics

The study was approved by the Regional Committee for Medical Research Ethics and the Norwegian Social Science Data Service. All participating patients signed an informed consent form.

### Statistical analysis

Data were compared between groups using a Chi-squared or Fisher’s exact test for dichotomous data, the Mann–Whitney for ordinal variables, and Student’s *t*-test for continuous variables. The paired-samples *t*-test was used to compare results scored before cardiac evaluation and at follow-up. Correlations were calculated as Pearson’s *r* (for pairs of continuous variables), point-biserial (for one dichotomous and one continuous variable), and phi (for two dichotomous variables). To identify which item or subset of items in BIPQ that best would predict Clinically significant complaints, a discriminant analysis procedure with a stepwise forward selection was run. In addition a series of logistic regression analyses were run, partly with forced entry of all eight items, partly with forward (conditional) inclusion and backward (conditional) elimination of independents. The best single item identified by these procedures subsequently was tested in combination with the remaining items (one at a time) to investigate the predictive properties of a minimal subset even further. All tests were two-tailed and p < .05 was considered significant. The Statistical Package for Social Sciences (SPSS) software was used (version 19; SPSS Inc., Chicago, IL, USA).

## Results

### BIPQ scores and changes from before to six months after the cardiac evaluation

Before the cardiac evaluation, patients with palpitations had a higher mean BIPQ score compared with those with chest pain on Items 1 (consequences), 5 (symptom frequency) and 7 (understanding). Otherwise, the groups did not differ significantly on any item before the cardiac evaluation or six months after (Table 2).

**Table 2:** **Comparison of the chest pain group (N =** **95) and the palpitationsgroup (N = 43) before and six months after negative cardiac evaluation**

**BIPQ (response options: 1 to 10)**	**Before cardiac evaluation**			**Six-month follow-up**		
	**Chest pain**	**Palpitations**	**p**	**Chest pain**	**Palpitations**	**p**
1. How much does your illness affect your life?	2.8 (2.1)	3.6 (2.5)	0.04	2.2 (2.0)	2.5 (2.0)	ns
2. How long do you think your illness will continue?	3.8 (2.5)	4.4 (3.0)	ns	4.8 (3.5)	4.8 (3.2)	ns
3. How much control do you feel you have over your illness?	4.0 (3.0)	4.2 (2.9)	ns	4.5 (3.0)	4.8 (3.1)	ns
4. How much do you think your treatment can help your illness?	5.3 (2.7)	5.4 (3.0)	ns	3.8 (2.9)	4.0 (3.1)	ns
5. How much do you experience symptoms from your illness?	3.1 (1.9)	4.1 (2.5)	0.04	2.5 (2.1)	2.7 (1.8)	ns
6. How concerned are you about your illness?	3.4 (2.1)	3.6 (2.3)	ns	2.7 (2.1)	2.9 (2.4)	ns
7. How well do you feel you understand your illness?	4.6 (3.0)	6.0 (2.6)	0.01	5.3 (3.0)	5.8 (3.0)	ns
8. How much does your illness affect you emotionally? e.g., does it make you angry, scared, upset, or depressed?	3.1 (2.4)	3.2 (2.6)	ns	3.0 (2.4)	3.1 (2.6)	ns

ns, not significant.Mean (SD) (Student’st-test).

The BIPQ scores of the total sample are presented in Table 3. At six-month follow-up, there were significantly lower scores than before the cardiac evaluation for Items 1 (consequences), 4 (treatment control), 5 (symptom frequency), and 6 (illness concern), while there was a significantly higher score for Item 2 (timeline). Items 3 (personal control), 7 (understanding), and 8 (emotional effect) did not change significantly (Table 3).

**Table 3:** **Scores on The Brief Illness Perception Questionnaire (BIPQ) measured before and six months after negative cardiac evaluation (N = 138) (Paired samples *t*-test)**

**BIPQ**	**Before cardiac evaluation** **Mean (SD)**	**Six-month follow-up** **Mean (SD)**	**Mean difference**	**p**
1. How much does your illness affect your life?	3.0 (2.3)	2.3 (2.0)	0.74	<.001
2. How long do you think your illness will continue?	4.1 (2.7)	4.8 (3.4)	−0.73	.01
3. How much control do you feel you have over your illness?	4.0 (3.0)	4.6 (3.0)	−0.60	.07
4. How much do you think your treatment can help your illness?	5.4 (2.7)	3.8 (3.0)	1.54	<.001
5. How much do you experience symptoms from your illness?	3.4 (2.1)	2.6 (2.0)	0.83	<.001
6. How concerned are you about your illness?	3.5 (2.2)	2.8 (2.2)	0.72	<.001
7. How well do you feel you understand your illness?	5.0 (2.8)	5.4 (3.0)	−0.42	.2
8. How much does your illness affect you emotionally? e.g., does it make you angry, scared, upset, or depressed?	3.1 (2.4)	3.1 (2.5)	0.01	.9

*p < .05.

**p < .01.

### Correlations between items of BIPQ and BDI and SF-36 scores before and six months after the cardiac evaluation

The correlations between scores of the BIPQ and BDI and SF-36 before cardiac evaluation and at follow-up are presented in Table 4. For almost all items the correlations were stronger at follow-up (Table 4).

**Table 4:** **Correlations between items of The Brief Illness Perception Questionnaire (BIPQ) and outcome measures (BDI and SF-36) rated at the same time (N = 138)(Pearson’s correlation coefficient *r*)**

	**Mean (SD)**	**BIPQ1** **consequences**	**BIPQ2** **timeline**	**BIPQ3** **personal control**	**BIPQ4** **treatment control**	**BIPQ5** **symptom frequency**	**BIPQ6** **illness concern**	**BIPQ7** **understanding**	**BIPQ8** **emotional effect**
BDI-1	5.1 (5.6)	.52**	.23**	–.05, ns	.16, ns	.31**	.39**	–.05, ns	.51**
BDI-2	5.8 (6.3)	.64**	.28**	–.31**	.28**	.63**	.47**	–.14, ns	.62**
SF-36									
Physical-1	65 (25)	–.24**	–.23**	.01, ns	–.22**	–.20*	–.16, ns	.09, ns	–.19*
Physical-2	69 (23)	–.50**	–.18*	.32**	–.21*	–.50**	–.31**	.15, ns	–.41**
Mental-1	73 (25)	–.41**	–.05, ns	–.06, ns	–.06, ns	–.18*	–.28**	–.10, ns	–.38**
Mental-2	74 (22)	–.54**	–.30**	.23**	–.15, ns	–.51**	–.47**	.18*	–.49**

1- before cardiac evaluation.

2- six-month follow-up.

ns, not significant.

* p < .05.

** p < .01.

### Items of the BIPQ as predictors of poor outcome

The correlation analyses for items in the BIPQ scored before the cardiac evaluation, and Clinically significant complaints, depression, and HRQOL six months later, are presented in Table 5. The questions about consequences (Item 1) and emotional effect (Item 8) were most strongly correlated with Clinically significant complaints six months later. A discriminant analyses with stepwise forward inclusion of independents as well as a series logistics regressions clearly indicated that Item 1 (asking about the consequences of the complaints) used as a stand-alone is the best screening tool for predicting Clinically significant complaints at follow-up. The optimal cut-off for Item 1 is ≥4, yielding a sensitivity of 51%, a specificity of 85%, a positive predictive value (PPV) of 71%, a negative predictive value (NPV) of 69%, and an OR = 6.5 (*r* = .38, p < .001). Combining Item 1 with one or more of the other items of BIPQ did not improve the scale’s predictive power significantly.

**Table 5:** **Correlations between items of The Brief Illness Perception Questionnaire (BIPQ) rated before the cardiac evaluation and Clinically significant complaints (0** **= no, 1 =** **yes), BDI, and SF-36 rated six-month follow-up (N** **= 138) (Pearson’s correlationcoefficient *r *and point-biserial)**

	**BIPQ1** **consequences**	**BIPQ2** **timeline**	**BIPQ3** **personal control**	**BIPQ4** **treatment control**	**BIPQ5** **symptom frequency**	**BIPQ6** **illness concern**	**BIPQ7** **understanding**	**BIPQ8** **emotional effect**
Clinically significant complaints	.41**	.22*	–.10, ns	.10, ns	.34**	.33**	–.01, ns	.28**
BDI	.45**	.25**	–.08, ns	.18*	.30**	.38**	–.05, ns	.47**
SF-36								
Physical	–.28**	–.17*	.12, ns	–.06, ns	–.23**	–.15, ns	.02, ns	–.27**
Mental	–.46**	–.26**	.09, ns	–.12, ns	–.35**	–.34**	–.02, ns	–.43**

ns, not significant.

*p < .05.

**p < .01.

## Discussion

The patients with benign palpitations reported more symptoms (Item 5), were more affected by the symptoms (Item1), but also had more understanding (Item 7) of the symptoms before the cardiac evaluation compared with the non-cardiac chest pain group, otherwise the groups had similar illness perception scores before the cardiac evaluation and 6 months later. Despite the decreases in the scores for the total group regarding consequences (Item 1), symptom frequency (Item 5), and illness concern (Item 6), the score for emotional effect (Item 8) did not change significantly. The score for understanding (Item 7) had not increased significantly six months after the cardiac evaluation. The items covering consequences (Item 1), symptom frequency (Item 5), concern (Item 6), and emotional effect (Item 8) were most strongly correlated with the BDI and SF-36 scores. These correlations were stronger at follow-up compared with those measured before the cardiac evaluation. The score of BIPQ Item 1: “How much do your complaints affect your life?” before the cardiac evaluation gave a reasonably accurate prediction of Clinically significant complaints six months later.

The patients in the present study reported fewer symptoms at follow-up than before the cardiac evaluation (decrease in Item 5: “How much do you experience symptoms from your illness?”). The reduction in reported consequences (Item 1) and illness concern (Item 6) might be a consequence of the reduction in symptoms. The reduced faith in treatment (Item 4) and the increase in rating of how long they thought their complaints would continue (Item 2) might be a logical consequence of not being offered specific treatment and the experience of sustained complaints.

Even though the patients reported fewer symptoms (Item 5), they were equally emotionally affected by the illness at follow-up (Item 8). This is consistent with a previous report from the same sample describing an increase in fear of bodily sensations and depression from before the cardiac evaluation to six-month follow-up [5], and with a study by McDonald et al. [8] noting that patients worry more about their heart after than before a normal cardiac evaluation. Furthermore, the patients in the present study did not report significantly more understanding (Item 7) or control (Item 3) of the illness even though all of them had been through a cardiac evaluation and during this process had spoken to a cardiologist (all chest pain patients) or to a specialized cardiac care nurse (all patients with palpitations). This is in line with the results of the study by Robertsen et al. who investigated this issue before cardiac evaluation and at two-week and two-month follow-up for patients with non-cardiac chest pain [14]. These findings are thought-provoking and point to the need for more focus and research on the consequences of a normal cardiac evaluation. It should lead to more specific information about non-cardiac conditions to increase the sense of security for these patients.

Items about how much the illness affected patients’ life (Item 1), how often they experienced symptoms (Item 5), how concerned they were about the illness (Item 6), and how much they were emotionally affected by the illness (Item 8) were most strongly correlated with depression and HRQOL. Surprisingly, the correlations between items of BIPQ and depression and the HRQOL were substantially stronger at follow-up. This finding indicates that, during the evaluation for cardiac disorders, these patients are getting more focused on their symptoms, and that the interpretation of the symptoms appear to be more closely linked to how they evaluate their well being after the cardiac evaluation. This might be an unintended effect of normal cardiac evaluation and might contribute to the tendency towards poor outcomes among these patients. Strategies to avoid such developments among patients should be highlighted.

The items about consequences (Item 1), symptom frequency (Item 5), illness concern (Item 6), and emotional effect (Item 8) were most strongly correlated with depression and HRQOL six months later. In the study by Donkin et al. [18], the same items of BIPQ were the strongest predictors of low reassurance one month after a negative exercise stress test. Because most of the models that aim to explain the development of medically unexplained symptoms emphasize frightening misinterpretations as a main reason [23], this match of items, which predicted reassurance and health (Clinically significant complaints, depression and HRQOL), is as expected.

The question about consequences (Item 1), with a cut-off point at ≥4, had rather good properties in screening for Clinically significant complaints six months later. Compared with the 21-item BDI (with a set point at ≥5) used in the same sample with the same dependent variable (Clinically significant complaints) [5], the PPV, specificity, and OR values were better for the one-item questionnaire (71% vs 66%, 85% vs 74%, 5.7 vs 5.2, respectively) but the sensitivity and NPV were lower (51% vs 64% and 69% vs 73%, respectively). Such a one-question screening instrument is easier to implement in a cardiac setting than the 21-item BDI. Therefore, we recommend the use of Item 1 in the BIPQ as a simple screening instrument during cardiac evaluation.

Non-cardiac chest pain and benign palpitations differ from most physical disorders by the unexplained nature of the symptoms and the lack of treatment. Items 3 (personal control), 4 (treatment control) and 7 (understanding) will perhaps be only vaguely understood by the patients as relating to these conditions. Our study supports this assumption by revealing weaker correlation with other health assessments both before and after the cardiac evaluation (Table 4) and lack of significant prediction of outcome for these items (Table 5). Items 1 (consequences), 5 (symptom frequency), 6 (illness concern) and 8 (emotional effect) showed strong correlations with other health assessments and rather good prediction of outcome. We suppose this might be the case for other medically unexplained symptoms as well, and this information should be emphasized when these patients are received in the health care system. Treatment should emphasize on increasing personal control and understanding in an attempt to help patients to handle their complaints in a better way. Illness perceptions seem to be valuable in the understanding of the mechanisms behind poor outcome for these patients and also valuable when focus for treatment is decided upon.

### Strengths and limitations

A major strength of this study is that it was performed in a general cardiac setting and included consecutive patients in a known catchment area. As for limitations, the assessment of Clinically significant complaints at follow-up was obtained by questionnaires developed for the present study and have not been tested independently for psychometric properties. There were some dropouts (10%) at follow-up. We speculate that patients, who were worried about their health and/or experienced sustained complaints, were more interested in participating in the follow-up investigation, and this might have led to a systematic loss of subjects. Internal consistency between variables tends to get stronger when questionnaires are repeatedly administered to the same respondents (learning effect), thus probably contributing to some of the observed increases over time in most correlations.

## Conclusions

Patients with non-cardiac chest pain and patients with benign palpitations had almost similar perception of their symptoms. BIPQ consisting of eight different items regarding illness perception, contributed to clarify the mechanisms behind poor outcomes. The BIPQ item “How much does your illness affect your life” showed reasonably good properties to predict outcomes six months later.

## Abbreviations

HRQOL: Health-related quality of life; MI: Myocardial infarction; BIPQ: Brief Illness Perception Questionnaire; BDI: Beck Depression Inventory; SF-36: SF-36 Health survey; PPV: Positive predictive value; NPV: Negative predictive value; OR: Odds ratio; R: Correlation coefficient; p: p-value.

## Competing interests

The authors declare that they have no competing interests.

## Authors' contributions

EJ conceived the study, participated in the design of the study, carried out data collection, performed the statistical analysis and drafted the manuscript. EWM conceived the study, participated in the design of the study and reviewed the manuscript. GM conceived the study, participated in the design of the study and reviewed the manuscript. TM participated in the design of the study, performed the statistical analysis and reviewed the manuscript. TD conceived the study, participated in the design of the study and reviewed the manuscript. All authors read and approved the final manuscript.
